# Allergen Exposure in Lymphopenic Fas-Deficient Mice Results in Persistent Eosinophilia Due to Defects in Resolution of Inflammation

**DOI:** 10.3389/fimmu.2018.02395

**Published:** 2018-10-30

**Authors:** Caroline M. Ferreira, Jesse W. Williams, Jiankun Tong, Crystal Rayon, Kelly M. Blaine, Anne I. Sperling

**Affiliations:** ^1^Section of Pulmonary and Critical Care Medicine, Department of Medicine, University of Chicago, Chicago, IL, United States; ^2^Committee on Molecular Pathology and Molecular Medicine, University of Chicago, Chicago, IL, United States; ^3^Department of Pathology, University of Chicago, Chicago, IL, United States; ^4^Committee on Immunology, University of Chicago, Chicago, IL, United States

**Keywords:** Th1/Th2 cells, eosinophils, apoptosis, lung, inflammation, asthma, lymphopenia

## Abstract

Asthma is characterized by chronic airway type-2 inflammation and eosinophilia, yet the mechanisms involved in chronic, non-resolving inflammation remain poorly defined. Previously, our group has found that when Rag-deficient mice were reconstituted with Fas-deficient B6 LPR T cells and sensitized and challenged, the mice developed a prolonged type-2-mediated airway inflammation that continued for more than 6 weeks after the last antigen exposure. Surprisingly, no defect in resolution was found when intact B6 LPR mice or T cell specific Fas-conditional knockout mice were sensitized and challenged. We hypothesize that the homeostatic proliferation induced by adoptive transfer of T cells into Rag-deficient mice may be an important mechanism involved in the lack of resolution. To investigate the role of homeostatic proliferation, we induced lymphopenia in the T cell-specific Fas-conditional knockout mice by non-lethal irradiation and sensitized them when T cells began to repopulate. Interestingly, we found that defective Fas signaling on T cells plus antigen exposure during homeostatic proliferation was sufficient to induce prolonged eosinophilic airway inflammation. In conclusion, our data show that the combination of transient lymphopenia, abnormal Fas-signaling, and antigen exposure leads to the development of a prolonged airway eosinophilic inflammatory phase in our mouse model of experimental asthma.

## Introduction

Asthma is a heterogeneous airway disease characterized by variety of clinical phenotypes. One of the most common phenotypes exhibited by patients, atopic asthma, is characterized by the presence of T helper type 2 (Th2) cells and the persistence of airway inflammation. Why asthmatic patients develop chronic inflammatory responses in their airways and lungs remains an unresolved question in pulmonary medicine. Determining the mechanisms involved in the perpetuation of respiratory inflammatory events will allow for a better understanding of the chronic nature of atopic disease.

Although apoptosis may provide a mechanism for the removal of activated T-cells in healthy individuals, studies have suggested that this process may be delayed in the airways of asthmatics (1). First, it has been found that the percentage of apoptotic lymphocytes in induced sputum was significantly decreased in patients with asthma compared with healthy controls (2). Second, asthmatics demonstrated increased numbers of cells positive for the anti-apoptotic molecule Bcl-2 compared to normal control subjects, and the expression of Bcl-2 correlated with severity of asthma (3, 4). Third, low expression of Fas mRNA and surface Fas receptor on pulmonary CD3^+^ T cells has been associated with persistence of inflammatory cellular infiltrates in asthmatic airways (5), and peripheral blood leukocytes from asthmatics have been shown to be less sensitive to Fas-mediated apoptosis (6). Finally, murine studies suggest that the persistence of eosinophils during Th2 airway inflammation is due to defects in apoptosis in these cells (7–10). Together, these studies suggest that understanding the role of apoptosis pathways may be a valuable avenue of study for atopic asthma.

Allergen-specific Th2 cells can be induced in mice, and they promote airway inflammation characterized by eosinophilia, goblet cell hyperplasia, and airway constriction. However, the animal models generally do not lead to prolonged inflammation unless there is either continued antigen-exposure for long periods of time or when there are specific genetic alterations in the mice. For instance, transgenic mice that over-express IL-5 (11), IL-9 (12), IL-11 (13), or IL-13 (11), or mice that are deficient for T-bet, all develop a spontaneous chronic Th2-type lung inflammatory disease that presents with eosinophilic inflammation, collagen deposition in the airways, and airway hyper-reactivity to methacholine challenge (14). These animal models of airway inflammation are spontaneous and do not involve any antigen exposure. In our previous studies, we investigated the role of a cell surface death receptor Fas (CD95), and its ligand (FasL) in the resolution of airway inflammation. Using adoptive transfer of either B6 or B6.LPR T cells into *Rag*^−/−^ mice, we found that Fas-deficiency on T cells led to a delay in resolution and the development of a prolonged inflammatory response. Mice that received wild type T cells resolved their acute allergen-induced inflammation within 2 weeks following the last challenge, while mice that received Fas-deficient T cells developed a prolonged inflammatory phase that lasted at least 4 weeks longer (15). In a follow-up study using adoptive transfer models, we also demonstrated that FasL-deficiency (GLD) on T cells led to prolonged airway inflammation (16). Thus, our murine model of prolonged Th2-mediated airway inflammation is unusual since it develops due to a failure to resolve an acute response, and not due to chronic allergen challenges or genetic manipulation.

Using a T cell-specific conditional deletion of Fas, we were able to address whether homeostatic proliferation was involved in the prolonged inflammatory phase. Interestingly, we found that these mice failed to induce the prolonged airway inflammation and eosinophilia after sensitization and challenge. Inducing homeostatic proliferation in these conditional T cell specific Fas-deleted mice restored the delayed resolution. These findings show a new potential mechanism involved in eosinophilic airway inflammation that could be involved in human asthma as well.

## Materials and methods

### Animals

C57BL/6 (B6) mice were purchased from The Division of Cancer Treatment at the National Cancer Institute (Frederick, MD). B6.129S7-Rag1^tm1Mom^ (Rag^−/−^) and B6.MRL-Tnfrsf6^LPR^ (B6.LPR) mice were purchased from The Jackson Laboratory. Fas^fl/fl^ lck-cre mice were a kind gift from Dr. Alexander V. Chervonsky (17). All animals were bred and housed within a specific pathogen-free barrier facility maintained by the University of Chicago Animal Resources Center. The studies reported here conform to the principles outlined by the Animal Welfare Act and the National Institutes of Health guidelines for the care and use of animals in biomedical research. All animal procedures and housing were approved by the University of Chicago Animal Resources Center and the Institutional Animal Care and Use Committee.

### Antibodies and flow cytometry

Anti-mouse CD3 (clone 17A2; BD Biosciences, San Diego, CA), anti-mouse CCR3 (clone 83101.111; R&D Systems, Minneapolis, MN), and anti-mouse Ly6G (GR1, BD Biosciences) antibody were used for flow of bronchial alveolar lavage (BAL) T cells (CD3^+^ side scatter low) and eosinophils (CCR3^+^Ly6G^−^ and side scatter high). Data was acquired on an LSR-II (Becton-Dickinson, San Jose, CA), and analyzed using FlowJo software (Treestar).

### *S. mansoni* sensitization and challenge

These methods were previously described (15, 18). Briefly, at day-14, mice were immunized by intraperitoneal (i.p.) injection of inactivated *S. mansoni* eggs. At days-7 and 0, the mice were challenged with 5 μg of soluble egg antigen (SEA) by intranasal and intratracheal aspiration, respectively. The mice were sacrificed at 4, 14, 21, or 28 days after the last challenge. B6.LPR mice were used at 5–7 weeks of age in order to ensure that they had not yet developed lymphoproliferative disease. For some experiments, the mice were irradiated with 6 Gy, 6 days prior to the *S. mansoni* sensitization.

### BAL analysis

Bronchioalveolar lavage (BAL) was performed by delivering ~0.8 ml of cold PBS into the cannulated trachea and gently aspirating the fluid. The lavage was repeated a total of four times, and a total volume of 2.5–3 ml BAL was collected. The percentage of cell types found within BAL fluid was determined by flow cytometric analysis with cell type–specific markers.

### Adoptive transfer

B6 and B6.LPR T cells were harvested from lymph nodes of donor mice and enriched by non-adherence to a nylon wool column. 10^7^ cells were adoptively transferred intravenously into each recipient. The purity, as determined by flow cytometry, was between 90 and 95% CD3^+^ T cells.

### Analysis of lung histological changes

Lungs were removed from mice after completion of BAL and fixed by immersion into 4% paraformaldehyde. Lobes were sectioned sagittally, embedded in paraffin, cut into 5 μm sections, and stained with H&E for analysis.

### Detection of Th1 and Th2 cytokines

T cells were incubated at a concentration of 4 × 10^6^ cells/ml in a 48-well plate which was pre-coated with αCD3 antibody (145-2C11). Supernatants were harvested after 48 h in culture. Cytokine production was measured using a Millipore Multiplex bead array following manufacturer's instructions and analyzed by Luminex (Bio-Rad) reader.

### Statistical analysis

Graph generation and statistical analysis were performed using Prism software (version 5.0; GraphPad). Differences in total cells and eosinophils in the BAL fluid and in lung histological scoring were determined by using an unpaired Student's two-tailed *t*-test. If the *F*-test differed significantly, Mann-Whitney tests were used. Error bars represent SEM. Statistical significance was claimed whenever ^*^P < 0.05; ^**^P < 0.01; ^***^P < 0.001.

## Results

### A prolonged inflammatory phase is not observed in B6.LPR mice

**Figure 1:**
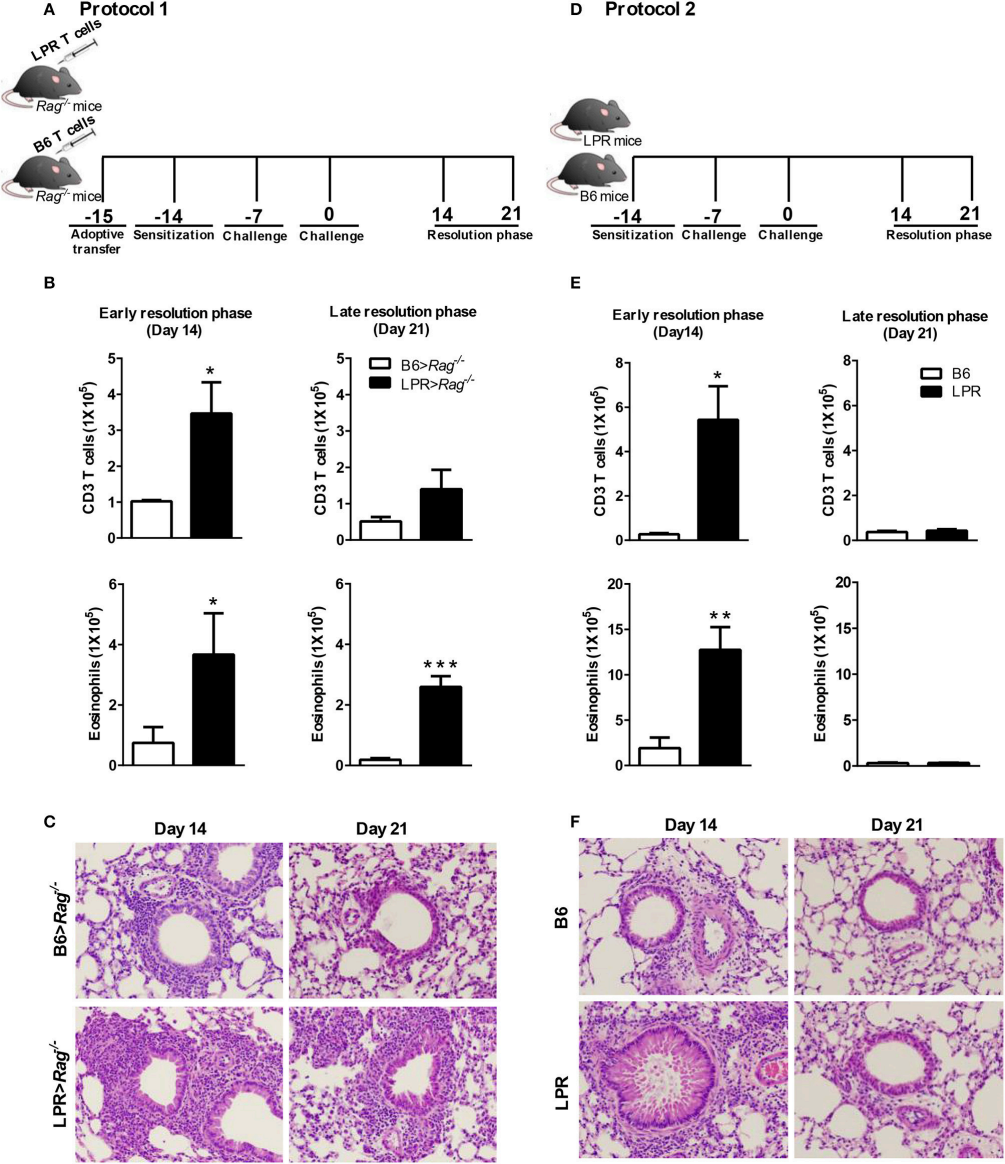
Fas signaling is required for normal resolution of eosinophilic airway inflammation. B6 and B6.LPR T cells were adoptively transferred into Rag^−/−^ mice 1 day before sensitization (noted as B6>Rag^−/−^ and LPR>Rag^−/−^ mice, respectively). The reconstituted mice were sacrificed on days 14 or 21 after the final challenge, and the BAL was analyzed **(A,B)**. B6 and B6.LPR mice were also sensitized and sacrificed on days 14 or 21 after the final challenge, and the BAL was analyzed **(D,E)**. Representative H&E stained sections of lungs at days 14 and 21 **(C,F)** Lung tissues from B6>Rag^−/−^, LPR>Rag^−/−^, B6, and LPR mice were fixed in 4% paraformaldehyde and embedded in paraffin. Approximately five mice per group per time point were analyzed. *P < 0.05. **P < 0.01. ***P < 0.001. Error bars represent SEM.

Using a murine model of Th2-mediated airway inflammation (15, 18), we have previously demonstrated an airway disease with several important similarities to allergic asthma. This protocol (Figure 1A) involves sensitizing mice with inactive *S. mansoni* eggs by intraperitoneal injection (i.p) and challenging through intratracheal administration with soluble eggs antigen (SEA) antigen. This protocol leads to a robust Th2 response, consisting of 70–75% of eosinophils, 10–15% of T cells, and 10–15% macrophages, at the peak of inflammation on day 4 after the last challenge. By 14 days after the last challenge (early resolution phase), the airway inflammation is almost fully resolved in WT B6 mice.

Consistent with our previous publication (15), we find that when T cells from B6.LPR (Fas-deficient) mice are transferred into a Rag^−/−^ host, then sensitized and challenged, the animals were unable to resolve their airway inflammatory response at similar rates to Rag^−/−^ animals receiving control B6 T cell transfers (Figure 1B). Elevated T cell and eosinophil numbers were found at 14 and 21 days post challenge (Figure 1B), as well as elevated overall inflammation (Figure 1C). Our previous study had shown similar responses at the peak of inflammation suggesting that the prolonged inflammation observed at later time points was not simply a more robust acute phase response, but a defect in resolution associated with Fas-deficiency on the T cells (15). It remained untested whether B6.LPR animals themselves would show similar defects in resolution of allergic airway inflammation. Following sensitization and challenge (Figure 1D), B6.LPR mice responded similarly at day 14 post challenge to the T cell adoptive transfer model, with elevated BAL T cells and eosinophils (Figure 1E). Surprisingly, unlike the adoptive transfer model (15), we found that B6.LPR mice had resolved their airway inflammation by day 21 after challenge (Figures 1E,F). These data suggest that Fas-deficiency is not sufficient to induce the prolonged inflammatory phase.

### Conditional deletion of Fas on T cells does not affect the resolution of inflammation

**Figure 2:**
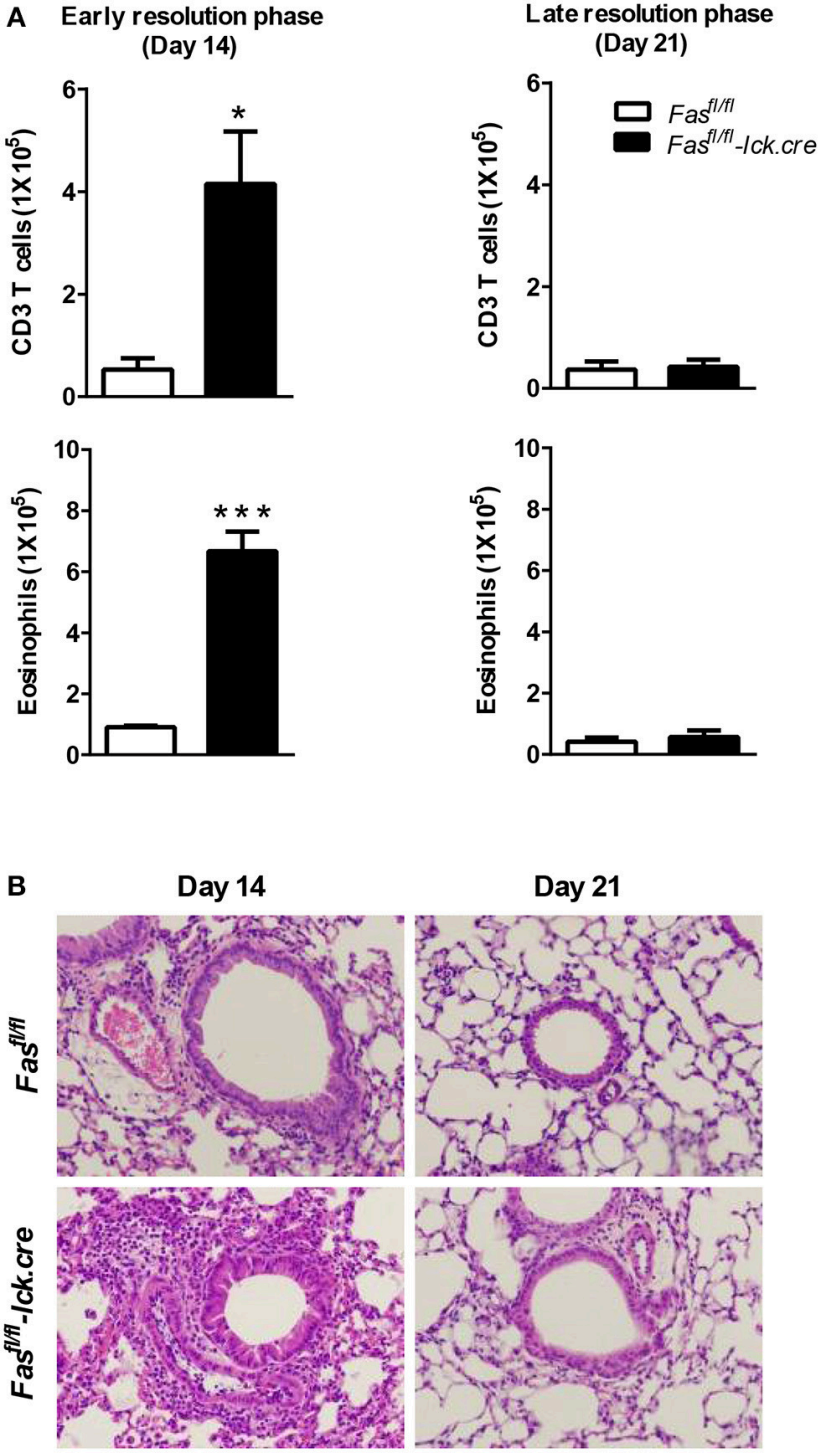
T cell-specific Fas signaling is sufficient to resolve early eosinophilic inflammation. Fas^fl/fl^ lck-cre and Fas^fl/fl^ (control mice) were sensitized and sacrificed on days 14 and 21 after the final challenge and the BAL was analyzed **(A)**. Representative H&E stained sections of lungs at days 14 and 21 **(B)**. Lung tissues from mice were fixed in 4% paraformaldehyde and embedded in paraffin. Approximately five mice per group per time point were analyzed. *P < 0.05. ***P < 0.001. Error bars represent SEM.

To address the possibility that deletion of Fas on T cells specifically could regulate the resolution of airway inflammation, we obtained Fas-conditional knockout mice (Fas^fl/fl^) and bred them to the T cell-specific Lck-cre mice. Fas^fl/fl^ lck-cre mice were sensitized and challenged following protocol 2 (Figure 1D) and assessed for inflammatory cell infiltrates in the airways. Fas^fl/fl^ lck-cre mice developed normal numbers of airway eosinophilia, lymphocyte infiltration, as well as vascular and peribronchial inflammation at day 4 after challenge as compared to littermate controls (Fas^fl/fl^) (data not shown). At 14 days after challenge, the Fas^fl/fl^ lck-cre mice showed elevated airway inflammation and tissue inflammation compared to littermate controls (Figure 2). Thus, Fas^fl/fl^ lck-cre mice have a similar delay in the resolution of airway inflammation as observed in B6.LPR and B6.LPR>Rag^−/−^ mice at the early resolution phase. However, the Fas^fl/fl^ lck-cre mice showed complete resolution of inflammation by day 21 after sensitization and challenge. These data suggest that while T cell-specific Fas signaling is required for effective resolution of inflammation during the early phase (day 14), the lack of Fas signaling on T cells specifically is not sufficient for the prolonged inflammation seen in the T cell transfer models.

### Antigen exposure during lymphopenia in T cell-specific Fas-conditional knockouts leads to development of persistent eosinophilia

By adoptively transferring T cells into lymphopenic recipients such as *Rag*^−/−^, T cells will undergo homeostatic proliferation to fill the open niche (19). This lymphopenia-induced proliferation is also well-documented in humans. Homeostatic proliferation of T cells occurs in humans during immune system development, as a mechanism to maintain T cell memory, and after transient lymphopenia during certain viral infections (20, 21). The role of lymphopenia has not been investigated as a factor in asthma pathology. Since we observed the development of prolonged airway inflammation only in a model in which Fas-deficient T cells are transferred into a lymphopenic host, we sought to test whether induction of lymphopenia in the intact *Fas*^*fl*/*fl*^ *lck-cre* or B6.LPR mice would induce the prolonged eosinophilia as previously found in the adoptive transfer model.

**Figure 3:**
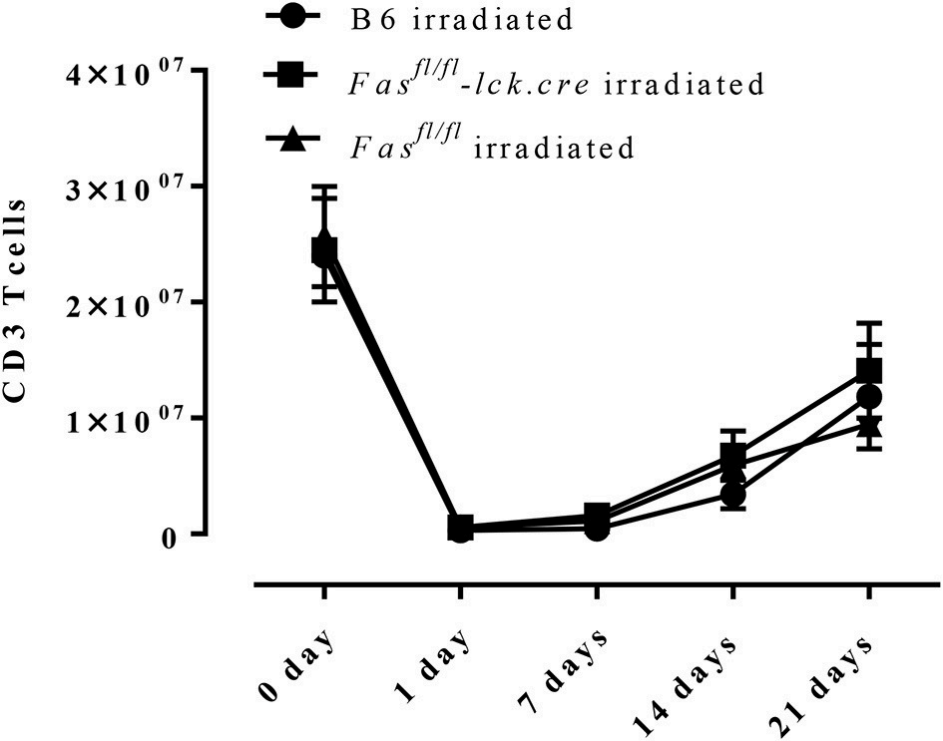
T cell recovery after irradiation-induced lymphopenia. B6 mice, Fas^fl/fl^ lck-cre, and Fas^fl/fl^ mice were irradiated with 6 Gy, CD3 T cells in the spleen were counted and analyzed by flow cytometry. Approximately 3–4 mice per group per time point (5-time points total) were analyzed.

**Figure 4:**
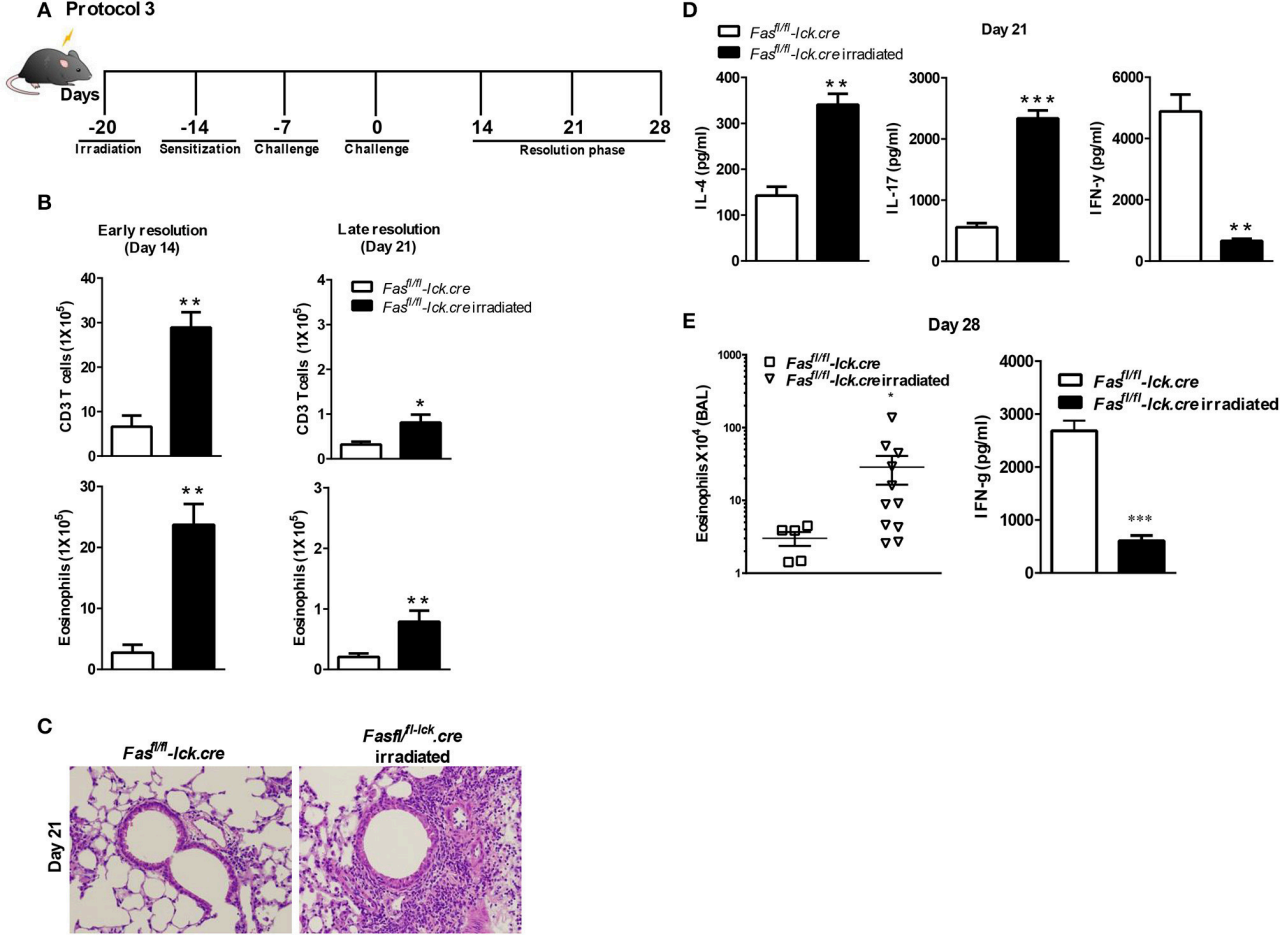
Antigen exposure during lymphopenia in T cell-specific Fas conditional knockouts leads to development of prolonged eosinophilic airway inflammation. *Fas*^*fl*/*fl*^ *lck-cre and Fas*^*fl*/*fl*^ *lck* (control mice) were irradiated (or not treated) with 6 Gy, 6 days prior to a *S. mansoni* sensitization, and sacrificed on day 14 or day 21 after the final challenge. The BAL was analyzed **(A** and **B)**. Representative H&E stained sections of lungs at day 21 **(C)**. Lung tissues from mice were fixed in 4% paraformaldehyde and embedded in paraffin **(C)**. Lung T cells from *Fas*^*fl*/*fl*^ *lck-cre and Fas*^*fl*/*fl*^ irradiated mice at day 21 were re-stimulated with anti-CD3 and measured by a Bioplex system as described in Material and Methods for IL-4, IL-17, and IFN-γ **(D)**. At day 28 post challenge, the eosinophil number in the BAL was evaluated **(E)** and IFN-γ by lung T cells. Approximately five-eleven mice per group per time point were analyzed. *P < 0.05. **P < 0.01. ***P < 0.001 Error bars represents SEM.

To address this hypothesis, we induced transient lymphopenia by sub-lethal irradiation (6 Gy) in B6, Fas ^fl/fl^, and Fas^fl/fl^ lck-cre mouse strains. In both Fas^fl/fl^ lck-cre and control Fas^fl/fl^ mice, we found that splenic lymphocytes were decreased 97% 24 h after irradiation, but after 6 days the numbers of lymphocytes began to recover and were almost completely normal by 21 days post irradiation (Figure 3). Thus, we sensitized Fas^fl/fl^lck-cre mice and control Fas^fl/fl^ littermates at 6 days after irradiation when homeostatic proliferation is occurring and challenged them as described in the protocol (Figure 4A). Sensitized and challenged lymphopenic Fas^fl/fl^ lck-cre mice developed normal levels of airway inflammation at day 4 after challenge whether or not they were irradiated (data not shown). However, the irradiated Fas^fl/fl^ lck-cre mice showed increased airway levels of T cells and eosinophils at days 14 and 21 compared to Fas^fl/fl^lck-cre mice that were not irradiated (Figures 4A,B). This was also readily observable in the cellular infiltration around airways and vessels in H&E sections (Figure 4C). Thus, Fas deficiency on T cells is sufficient to induce prolonged airway inflammation when the antigen exposure occurs during time periods when the T cells are undergoing homeostatic proliferation.

To determine the nature of the T cell response at 21 days that is inducing the prolonged inflammation, lung T cells were restimulated *in vitro* and cytokine production of IFN-γ, IL-10, IL-17, IL-4, IL-5, and IL-13 were measured. Interestingly, the level of IFN-γ production by irradiated Fas^fl/fl^lck-cre T cells was significantly less compared to non-irradiated T cells at day 21 (Figure 4D). These data are consistent with our previous findings that the failure of T cells to produce IFN-γ in the LPR>Rag^−/−^ mice plays an important role in the resolution of Th2 airway inflammation (15). However, levels of IL-4 and IL-17 were higher in irradiated Fas^fl/fl^lck-cre cells than T cells from non-irradiated Fas^fl/fl^lck-cre mice, consistent with increased levels of inflammation (Figure 4D). There were no differences in levels of IL-10 (Supplementary Figure 1), IL-13 and IL-5 between groups (data not shown). Finally, even at day 28 after the last challenge, we found that Fas^fl/fl^lck-cre irradiated animals had persistent eosinophilia and **decreased IFN-**γ **production by T cells** (Figure 4E). Cytokine production by T cells was also evaluated at this time point, and the irradiated Fas^fl/fl^lck-cre animals showed a reduction in IFN-γ levels with an increase in IL-13 (data not shown). Together, these data support a conclusion that T cells undergoing homeostatic proliferation rely on Fas for regulation of inflammatory responses.

### LPR mice develop prolonged type 2 inflammation when the sensitization occurs while T cells are undergoing homeostatic proliferation

**Figure 5:**
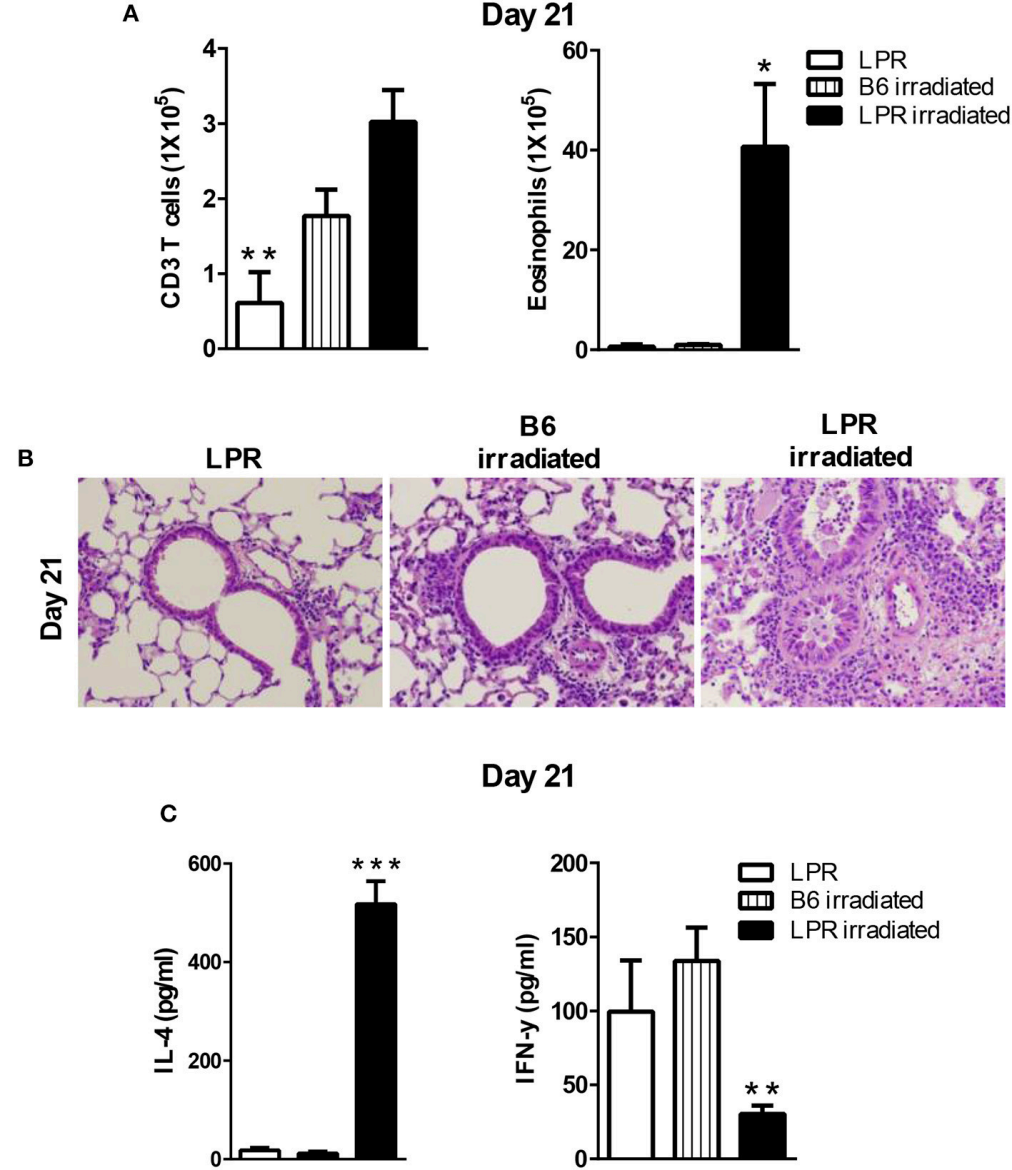
LPR mice are able to develop prolonged inflammation when the sensitization occurs during lymphopenia. B6.LPR and B6 and B6.LPR irradiated mice (6 Gy, 6 days prior to the *S. mansoni* sensitization) were sacrificed on days 14 or 21 after the final challenge and the BAL was analyzed **(A)**. Representative H&E stained sections of lungs at day 21 **(B)**. Lung tissues from mice were fixed in 4% paraformaldehyde and embedded in paraffin. **(C)** Lung T cells from B6.LPR and B6.LPR irradiated mice at day 21 were re-stimulated with anti-CD3 and measured by a Bioplex system as described in Material and Methods for IL-4 and IFN-γ. Approximately, five mice per group per time point were analyzed. *P < 0.05. **P < 0.01. ***p < 0.001. Error bars represent SEM.

Our data showed that B6.LPR mice do not sustain prolonged inflammation by day 21 after our sensitization and challenge protocol (Figure 1E). Similar to the irradiation protocol just described for the Fas^fl/fl^lck-cre mice, we sensitized B6.LPR mice and control mice (B6) at 6 days after irradiation, when homeostatic proliferation is occurring, and challenged them as described in protocol 3 (Figure 4). Irradiated B6.LPR mice had increased airway levels of total cells (data not shown) and eosinophils (Figure 5), while the number of T cells did not change significantly during the late resolution phase when compared irradiated B6 mice. Further, we observed an increased infiltrate of cells around the airways and vessels in the B6. LPR irradiated mice compared to irradiated B6 mice (Figure 5B).

To determine the nature of the T cell response that was induced during the prolonged inflammation, lung T cell cytokine production from restimulated cells was measured for IFN-γ, IL-10 IL-17, IL-4, IL-5, and IL-13. Interestingly, the levels of IFN-γ production were significantly less in the B6.LPR irradiated mice compared to non-irradiated B6.LPR mice (Figure 5C). We also found a significant difference in IL-4 between B6.LPR non-irradiated mice and B6.LPR irradiated mice at 21 days after the last challenge. B6.LPR irradiated mice produced significantly more IL-4 (Figure 5C) but the same levels of IL-17 (data not shown). These findings again suggest that IFN-γ production attenuates airway inflammation in a murine model of asthma, and these data are consistent with our previous findings that the failure of B6.LPR T cells to produce IFN-γ in adoptive transfer model plays an important role in the ability to resolve Th2-medicated inflammation.

## Discussion

**Figure 6:**
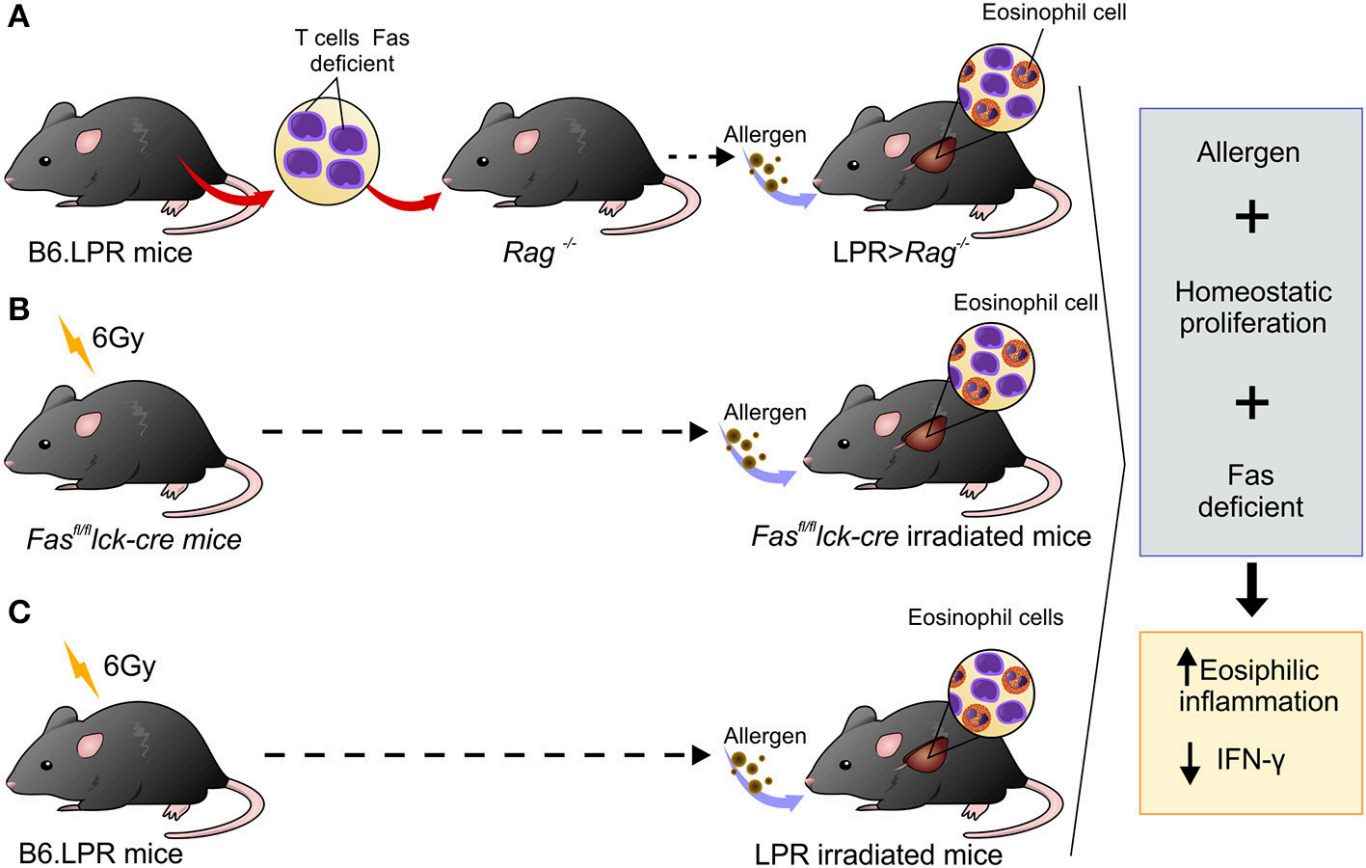
Schematic representation showing three different experimental designs leading to persistent eosinophilia in a mouse model. **(A)** B6.LPR T cells were adoptively transferred into *Rag*^−/−^ mice, **(B)** *Fas*^*fl*/*fl*^ *lck-cre* irradiated with 6 Gy and **(C)** B6.LPR irradiated with 6 Gy. All three situations involve transient lymphopenia, abnormal Fas-signaling, and antigen exposure resulting in decreasing levels of IFN-γ in the lung.

Many advances have been made in our understanding of factors involved in type 2 airway inflammation onset, but little is known about the mechanisms involved in the resolution process. Studies conducted in mice to investigate mechanisms associated with chronicity of eosinophilic inflammation have been carried out by exposing mice to continuous allergen challenges. However, the persistent lung inflammation observed in asthmatic patients cannot be explained by continuous allergen exposure (22). Thus, the objective of this investigation was to determine the possible mechanisms involved in the development of prolonged airway inflammation without continued exposure to allergen (15). The novelty of our study is that the combination of three factors, defective Fas-signaling on T cells, allergen exposure, and homeostatic proliferation, are all necessary to develop chronic Th2 inflammation (Figure 6). Thus, asthmatic patients may not only have hyperactivity of the Th2 immune response but may also have defects in their ability to resolve inflammation.

Lymphopenia is commonly associated with many viral infections including respiratory syncytial virus (RSV), influenza, measles, rubella, and parvovirus, West Nile virus and rhinovirus (RV) (23–27). Viral respiratory infections can have a profound effect on many aspects of asthma including its initiation, exacerbation, and severity (28–30). Interestingly, RSV and RV infections in children have been found to be an important risk factor for later development of asthma (31–35). Mechanisms linking RV infection and asthma development involve allergen exposure and host factors such as polymorphisms. Our data in a mouse model shows that homeostatic proliferation plays an important role in the development of persistent inflammation and eosinophilia. Thus, it is possible that lymphopenia is a part of the mechanism that associates RSV and other viruses with asthma risk.

Besides viral infection, it is also important to note that elevated rates of homeostatic proliferation are associated with some autoinflammatory syndromes, such as rheumatoid arthritis and systemic lupus erythematosus (10, 36). Chemotherapy and bone marrow transplantation can be associated with autoimmune manifestations (37). Although studies that correlate chemotherapy with asthma induction or severity are rare, unexplained asthma symptoms in patients receiving Tamoxifen for the treatment of breast cancer have been described (38).

Our data demonstrate that expansion of T cells in Lpr>*Rag*^−/−^ mice is not only due to homeostatic proliferation, but also requires specific antigen stimulation (data not shown). However, we have yet to determine whether polyclonal stimuli could replace homeostatic proliferation and play a role in the prolonged eosinophilia found in our model. Recently, it has been shown that recurrent T cell homeostatic proliferation results in global gene expression changes, including the progressive upregulation of FasL, granzyme B, and programmed cell death protein 1 (PD-1) (39). Considering that PD-1 is expressed during chronic T cell activation and recurrent T cell homeostatic proliferation, and considering that PD-1 expression on T cells inhibits IFN-γ production (40), it is possible that PD-1 may play a role on our model.

There is a possibility that a neutralizing Fas antibody treatment reproduces the same results observed in our model, considering that neutralizing Fas antibody may limit the expansion of antigen-specific T cells. Further, it has previously been reported that the administration of neutralizing antibody to FasL (clone MFL4) in Balb/c mice induce increased and persistent eosinophilia (10). However, in this case, the acute prolonged eosinophilia was resolved by day 10 after challenge, not during a chronic inflammatory phase as observed in our model. Further, this study used Balb/c and not C57BL/6 mice, and a different model of airway inflammation. Nevertheless, this paper and our data both suggest that Fas functions to dampen the acute and persistent lung inflammatory response characteristic of asthma.

The prolonged inflammatory phase observed in our experiments is correlated with decrease levels of IFN-γ production and increases in Th2 cytokines from lung T cells. After recovery from viral infections, reduced IFN-γ expression in PBMCs and airway cells has been observed and associated with both increased asthma risk and asthma severity (41, 42). Thus, it is important to note that the factors observed in our mouse model that led to prolonged Th2 inflammation, lymphopenia and decreased IFN-γ production, can also be observed during viral infections. IFN-γ promotes Fas-mediated apoptosis of allergen-activated T lymphocytes in the airways of atopic asthmatic patients (43). Other studies have shown that IFN-γ has a potent local and systemic effects through its actions on the airway epithelium in mice (44). Also, studies of inhaled IFN-γ in humans show that mild asthmatics that inhaled IFN-γ for 3 weeks exhibited a reduction in airway eosinophils (45). Further, IFN-γ has been shown to have a critical role in triggering T cell apoptosis via the up-regulation of Fas on the surface of activated CD4+ T cells (46). Finally, it has been shown that FasL activation exerts an inhibitory effect on IL-5, IL-9, and GM-CSF during the allergic airway responses in the BAL fluid of mice (10).

We find that IFN-γ production by T cells is reduced when either the *Fas*^*fl*/*fl*^*lck-cre* or LPR mice develop persistent airway inflammation past 21 days. We have previously transferred IFN-γ^−/−^ T cells into Rag^−/−^, using same approach as used in Figure 1A with LPR T cells in to the Rag^−/−^and our previous study of FasL-deficient (GLD) T cells into the Rag^−/−^(15). All these mice (IFN-γ^−/−^ 0 > *Rag*^−/−^, LPR>*Rag*^−/−^, and GLD>*Rag*^−/−^) develop chronic eosinophilic inflammation (15, 16). Since IFN-γ production by T cells is greatly reduced in the absence of Fas expression, these data suggest that IFN-γ plays an important role in the resolution of eosinophilic lung inflammation in our model. It is possible that there is an impaired apoptosis of Ag-specific Th2 cells in the lungs of these mice due to the decrease in IFN-γ induced apoptosis. The association between Fas and IFN-γ signaling leading to the resolution of eosinophilic airway inflammation needs to be carefully dissected to fully understand their relative contributions to allergic disease models.

Here, we focus on uncovering the role of homeostasis proliferation in the development of persistent inflammation observed in LPR> *Rag*^−/−^ mice. However, we did not assess other asthma characteristics such as airway hyper responsiveness (AHR). Our group has previously shown that Fas-deficient T cells, homeostatic proliferation and antigen (using LPR>*Rag*^−/−^ mice) have both persistent eosinophilia and airway hyper responsiveness to methacholine at day 42 (15). Although it is known that the pathways leading from eosinophilic inflammation may not be the same ones that cause changes in lung function (47, 48), data from our group show that both occur together in this chronic inflammation model.

In addition to IFN-γ, the sustained lung eosinophil inflammation observed in our model is also associated with a prolonged capacity of lung T cells to maintain IL-4 production. Mice deficient in the IL-4 gene are defective in their development and maintenance of Th2 cells, as well as their ability to attenuate allergic airway inflammation in murine models (44, 49). Besides IL-4, Fas^fl/fl^lck-cre irradiated mice had increased production of IL-17. These findings are in line with the literature that demonstrates that IFN-γ can inhibit Th17 differentiation (45). Interestingly, it has been shown that IFN-γ facilitates antigen-induced apoptosis of Th17 (50). Thus, in the future could be interesting to evaluate the role of IL-17 in the persistent inflammatory phase of our model.

There is increasing evidence that persistent and non-resolution of allergic inflammation pathways may be related to the poor functioning of pro-resolvins lipid mediators (such as lipoxins, resolvins, protectins, and maresins) (51). These mediators aren't immunosuppressive, but instead support the resolution of ongoing immune responses. For instance, Resolvin E1 (RvE1) is a potent mediator that promotes resolution of inflammatory airway responses. Interestingly, RvE1 regulates the production of IL-17 and IFN-γ by T cells (51). Thus, the role of resolvins in our persistent eosinophilic inflammation models needs to be investigate as RvE1 may be involved in the dysregulation of IFN-γ production by Fas-deficient T cells.

In summary, we have demonstrated that the combination of transient lymphopenia, abnormal Fas-signaling, and antigen exposure leads to the development of a prolonged airway inflammatory phase in our mouse model of experimental asthma. This phase is correlated with decrease levels of IFN-γ in the lung and increases in Th2 cytokines. Taken together, our findings suggest that in humans, transient lymphopenia, as well as features of the host such as genetic polymorphisms in Fas, are likely to significantly influence immune responses in the airways and lungs.

## Author contributions

AS, CF, and JW conceived and designed the experiments. CF, JW, CR, JT, and KB performed the experiments. AS, CF, and JW analyzed the data. AS contributed reagents, materials, analysis tools. AS, CF, and JW wrote the paper.

### Conflict of interest statement

The authors declare that the research was conducted in the absence of any commercial or financial relationships that could be construed as a potential conflict of interest.

## Supplementary material

Supplementary Figure 1 IL-10 did not differ between groups, *Fas*^*fl*/*fl*^ *lck-cre* and *Fas*^*fl*/*fl*^ *lck* irradiated at 21 days after last challenge. Lung T cells from *Fas*^*fl*/*fl*^ *lck-cre* and *Fas*^*fl*/*fl*^ *lck* (control mice) irradiated mice at day 21 after the final challenge were -re-stimulated with anti-CD3 measured by Bioplex system as describe in Material and Methods for IL-10. Approximately 4–5 mice per group were analyzed. Error bars represents SEM.
