# Supplementary figures and images for: Allergen Exposure in Lymphopenic Fas-Deficient Mice Results in Persistent Eosinophilia Due to Defects in Resolution of Inflammation
Source: Front Immunol. 2018 Oct 30;9:2395. doi: 10.3389/fimmu.2018.02395 (PMC6219400; doi:10.3389/fimmu.2018.02395)

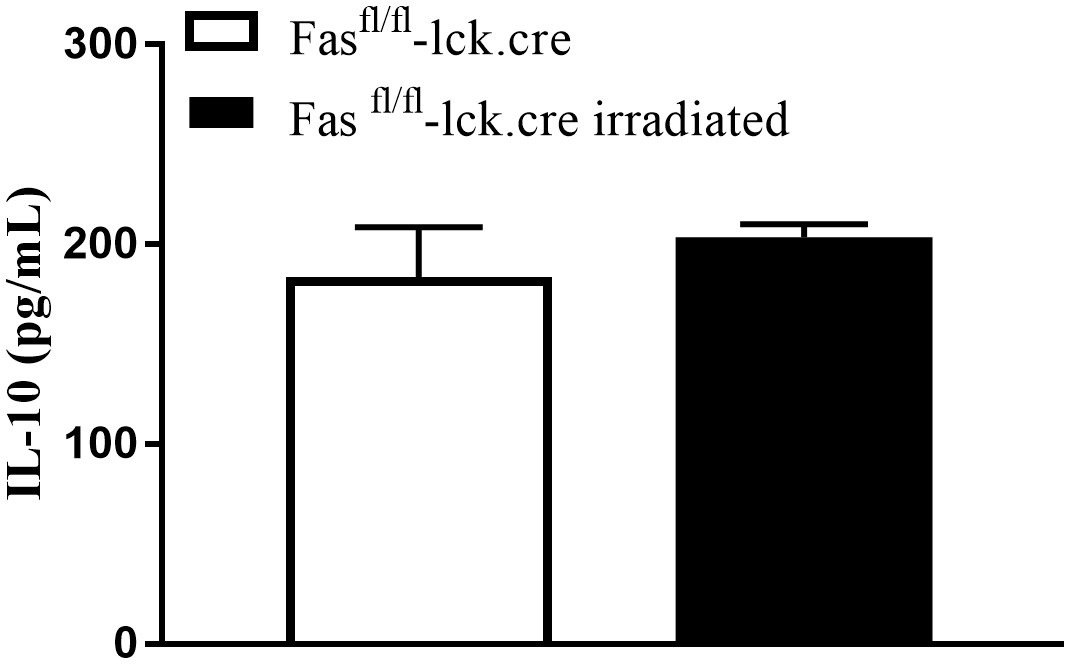

Supplement: Supplementary Figure 1 — IL-10 did not differ between groups, Fasfl/fl lck-cre and Fasfl/fl lck irradiated at 21 days after last challenge. Lung T cells from Fasfl/fl lck-cre and Fasfl/fl lck (control mice) irradiated mice at day 21 after the final challenge were -re-stimulated with anti-CD3 measured by Bioplex system as describe in Material and Methods for IL-10. Approximately 4–5 mice per group were analyzed. Error bars represents SEM. [file Image_1.TIF]
